# Hallucinations

**Published:** 1908-05-16

**Authors:** 


### Mental Diseases.

### HALLUCINATIONS.

An hallucination is usually described as a perception which has no objective cause, and is to be distinguished from an illusion, which is a false perception of a real object. In an hallucination the stimulus does not arise outside the body, but either in the end organ, or in the cerebral centre, or in the nervous tract between the two: it is thus a morbid process. In an illusion a sense organ is stimulated in the ordinary way by an object outside the body, and the stimulus is carried to the cerebral centre; but the intellect wrongly interprets the sensation. Both, it must be noticed, arise from the stimulation of a special sense. As an example, if a person looking into an empty street actually sees a black object go past when there is no object there, that is a visual hallucination; but if a piece of black cloth blows past, and the person believes that what he sees is a black dog, that is an illusion of sight. Amongst sane people hallucinations (except of the simplest kind) are comparatively uncommon, whilst the frequency of illusions is testified by the proverb, "All is not gold that glitters." It may be noted here that a delusion is a false belief—a purely intellectual process, and one not dependent on a special sense.

Hallucinations are met with of every special sense—of sight, hearing, taste, smell and common sensation; they are also described of muscular sense. They vary from vague and ill-defined to most vivid perceptions; but the essential feature of them all is their absolute reality or objectivity to the person who has them. The commonest ones are those of hearing, and the most uncommon those of smell. They are sometimes found to be unilateral, or to differ on the two sides; thus visual hallucinations may only occur in one eye, or auditory ones may differ in the right and left ears. Sometimes the auditory or visual hallucinations cease on closing the ears or eyes, but this is not always the case.

Hallucinations alone are not a sign of insanity; the simplest ones—*e.g.* flashes of light, ringings, buzzings, etc.—occur to most people at times, and more systematised ones to some who are perfectly sane. But though they do not alone indicate insanity, they very frequently accompany or cause it, and are of great importance as they so often give rise to delusions. In the early stages they are almost always indefinite, and become systematised with time.

Taking an auditory hallucination, as being the commonest, for an example, its life history would be probably as follows. The patient first experiences noises, which increase in frequency and intensity and for which he can assign no cause. With this increase of frequency and intensity the attention is more and more compelled to them; and the patient, not realising or being unable to believe their true meaning, owing to their absolute objectivity, begins to think out a cause for them. The imagination soon comes into full and unrestrained play, and the patient says he hears voices talking to him; the noises, with the help of the imagination, being transformed into words. At this stage he will not at first be able to tell exactly what the voices say nor who is talking, but a little later will say what they talk about, though unable to quote the exact words. Later still he will say who is speaking, and what are the exact words said, the delusion that some definite person is speaking to him having thus appeared. If the patient is able to quote the exact words he hears it can be taken for granted that the hallucinations have existed for some time. The process above described may stop short at any point with the cessation of the hallucinations; but if these do not cease it is easy to understand how delusions arise. Should the patient realise and believe that his sensations are due to subjective causes, he will not become deluded; but this appears to happen seldom owing to the intense objectivity of the hallucinations.

It is possible that hallucinations and the accompanying delusions may be of a harmless kind; but, unfortunately, experience teaches that the opposite is generally the case. The voices usually insult or otherwise goad to fury, at times causing the patient to strike someone, or otherwise to behave irrationally. Again, the voice may command the patient to do certain things, even commit suicide or murder, and if the voice is insistent, or is believed to be that of someone who must be obeyed—*e.g.*, the Almighty—the results may be disastrous. The tendency in hallucinated cases is always towards irresponsibility.

The following are examples of hallucinations giving rise to delusions in insane patients at present under care. A widow, whose mental attack commenced some years ago, after the death of her husband, suffers from vivid auditory hallucinations, which occur every night, and sometimes in the day. At times she has simultaneous hallucinations of every special sense—hears voices, sees visions, has evil smelling and tasting gases and powders pumped into her room, and is played on by electricity, whilst being alternately baked and frozen. Another lady believes she is verminous; she can feel lice crawling over her. She is scrupulously clean, but no amount of cleansing will remove the delusion produced by her hallucination of common sensation. A third case is persecuted by boys in the street at night. They throw filth into her room and fill her mouth with fæces. How this is done she cannot explain, but she knows that it happens, as she tastes and smells it. Yet another lady, a puerperal case, had a visual hallucination of the head and shoulders of a very beautiful woman. This hallucination seems to have appeared quite suddenly. It did not trouble her until one day it spoke, told her to do something, and she promptly obeyed; the result was terrible. For a long time she retained the visual hallucination, but has had no recurrence of the auditory one. She could describe the vision, and see it as she spoke to one. She knew that other people could not see it, though it was intensely real to her; but nothing can persuade her that the voice she heard was not real, or that it did not actually come from the beautiful vision.

